# Pathogenic variants in COL4A3, COL4A4, JAG1, and NPHS2 genes in focal segmental glomerulosclerosis: Insights from targeted gene panel sequencing

**DOI:** 10.1016/j.ymgmr.2026.101330

**Published:** 2026-06-15

**Authors:** Lava I. Ahmed, Dlnya A. Mohammed, Dana Ahmed Sharif

**Affiliations:** a Department of Biology, College of Science, University of Sulaimani, Sulaimani, Kurdistan Region, Iraq; b Department of Medicine, College of Medicine, University of Sulaimani, Sulaimani, Kurdistan Region, Iraq; c Nephrology Department, Shar Teaching Hospital, Sulaimani, Kurdistan Region, Iraq

**Keywords:** Focal segmental glomerulosclerosis, Targeted gene panel, Next-generation sequencing, COL4A3, COL4A4, JAG1, NPHS2, Pathogenic variants, Alport syndrome

## Abstract

**Background:**

Monogenic causes of focal segmental glomerulosclerosis (FSGS) are increasingly recognized, but data from highly consanguineous Middle Eastern populations remain limited. This study explored the diagnostic yield and descriptive genotype–phenotype correlations of a targeted gene panel in Iraqi patients with biopsy-proven FSGS from a cohort enriched for familial, early-onset, and consanguineous disease.

**Methods:**

Thirty consecutive patients with histologically confirmed FSGS underwent next-generation sequencing using a 98-gene renal disease panel. Variants were classified according to ACMG guidelines and interpreted with clinical and histopathological findings. Exploratory analyses compared variant-positive and variant-negative patients and assessed simple clinical predictors of a positive genetic result.

**Results:**

Pathogenic variants were identified in 10 of 30 patients (33.3%) in COL4A3 (*n* = 2), COL4A4 (*n* = 3), JAG1 (n = 3), and NPHS2 (n = 2). Two novel frameshift variants were detected in COL4A4 (c.3109_3110delCT) and JAG1 (c.1713delC). Variant-positive patients had earlier disease onset than variant-negative patients (20.6 ± 7.2 vs. 30.1 ± 11.6 years; *p* = 0.022). In this small, enriched cohort, a simple triage rule based on age of onset <25 years, extrarenal manifestations, or family history showed 100% sensitivity and negative predictive value, but requires external validation before clinical use. Gene-group analyses suggested collagen IV-related disease, recessive podocytopathy, and Alagille-spectrum disease in relevant subgroups.

**Conclusions:**

In this predominantly familial and early-onset FSGS cohort, one-third of patients harbored pathogenic variants, supporting the value of gene-panel testing in selected young or syndromic patients while underscoring the need for validation in larger, more representative cohorts.

## Highlights

• Targeted 98-gene sequencing identified pathogenic variants in 33.3% of Iraqi FSGS patients.
• COL4A3, COL4A4, JAG1, and NPHS2 accounted for all pathogenic variants detected.
• Two novel pathogenic frameshift variants were identified in COL4A4 and JAG1.
• Variant-positive patients had significantly earlier disease onset than variant-negative patients.
• Genetic testing refined diagnosis and identified collagen IV, NPHS2, and JAG1-related disease.

## Graphical abstract

**Figure:**
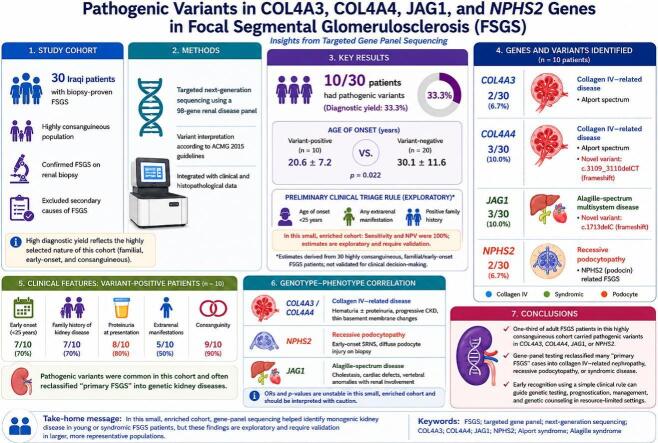

## 1 Background

Focal segmental glomerulosclerosis (FSGS) is a major cause of steroid-resistant nephrotic syndrome (SRNS) and end-stage renal disease (ESRD) worldwide [1], [2]. FSGS has shown a highly increased genetic heterogeneity with more than 60 monogenic genes identified to date that can cause isolated forms of FSGS and other podocytopathies [3], [4]. Understanding the genetic basis of FSGS is important for the diagnosis, prognosis, treatment and genetic counseling of FSGS patients [5].

The genetic diagnostic rate of FSGS varies greatly depending on the criteria used and has been reported to range from 10% to 40% using various clinical criteria including patient criteria, ethnic group, age at presentation and/or presence of a positive family history [5], [6]. The genetic variants in the type IV collagen genes (COL4A3, COL4A4, and COL4A5) that cause Alport syndrome were identified in up to 30% of sporadic and familial FSGS cases [7]. Similarly, mutations in podocyte genes such as NPHS2 (podocin) encoding protein, mutations are a well-defined cause of autosomal recessive FSGS [8], [9].

Consanguineous populations are an excellent source for identification of recessive disease-causing mutations and novel loci. Increased prevalence of consanguinity in a population increases the likelihood of sharing homozygous pathogenic variants thereby increasing the diagnostic yield [10], [11]. However, there are no genome-wide association studies or other large-scale genetic analyses to determine the spectrum of disease-causing mutations in FSGS mutations in different ethnic populations. Recent studies have confirmed that COL4A3–COL4A5 variants and other monogenic causes are frequent in adult-onset FSGS and chronic kidney disease, and have refined indications for genetic testing in FSGS patients, particularly those with early onset, family history, consanguinity, or extrarenal features.

We conducted targeted next-generation sequencing on a 98-gene panel in 30 biopsy-proven FSGS patients to evaluate the diagnostic yield and spectrum of mutations, to examine the genotype-phenotype association and to identify novel mutations.

## 2 Materials and methods

### 2.1 Study population and clinical evaluation

In this study, 30 patients with confirmed FSGS by biopsy were recruited from General Hospital and the private clinic in Kurdistan region, Northern Iraq. All patients had full clinical assessment that included medical history, full clinical examination and laboratory investigations. The criteria for inclusion of patients were confirmed diagnosis of FSGS by renal biopsy by two expert nephropathologists, availability of enough DNA for genetic analysis and informed consent. The criteria for exclusion of patients were secondary causes of FSGS such as obesity, drug-induced nephropathy and viral infections. Clinical information was derived from the patient records as follows: age at presentation, age of onset of disease, gender, family history of kidney disease (defined as chronic kidney disease or ESRD in first- or second-degree relatives), consanguinity, clinical features and investigations related to the underlying kidney disease (such as proteinuria, haematuria, hypertension, weight loss or headache), serum creatinine, blood urea nitrogen levels, severity of proteinuria, response to standard immunosuppressive therapy (e.g., steroid sensitivity or resistance when available) and need for renal transplantation and confirmation of specific FSGS variant on histopathology. For this study, age of onset was defined as the age at first documented clinical evidence of kidney disease (onset of persistent proteinuria and/or hematuria) on medical records. The study protocol was approved by the institutional review board and was conducted in accordance with the Declaration of Helsinki. Written informed consent was obtained from all participants or from their legally authorized guardians.

### 2.2 Genetic analysis

Genomic DNA was extracted from blood-derived leukocytes using standard DNA extraction protocols. Next-generation sequencing (NGS) was performed using an Applied Biosystems (ABI) 98 gene renal disease panel (Supplementary Table 1). Genes included are associated with FSGS, nephrotic syndrome, glomerular basement membrane disease as well as different types of ciliopathies (COL4A3, COL4A4, COL4A5, NPHS1, NPHS2, ACTN4, INF2, MYO1E, TRPC6, JAG1 and other ciliopathy-associated genes). The panel was designed using Ion AmpliSeq Designer (Thermo Fisher Scientific) to generate two primer pools that contain 2742 amplicons that were then sequenced on an Ion S5 platform using an Ion 540 chip as per manufacturer instructions. Paired-end sequencing reads were analyzed using the Ion Torrent workflow. Reads were mapped to the human reference genome (GRCh37/hg19) and variants were called and annotated using the Ion Reporter software (Thermo Fisher Scientific) and further analyzed using the Franklin platform (Genoox). For the analysis, variants with a depth of coverage >20× and a base call quality of Q > 30 were selected. Variants with frequencies above 1% in the gnomAD database were excluded. Coding variants (missense/nonsense/frameshift/splice-site) and non-coding variants predicted to have regulatory function were prioritized for further analysis.

### 2.3 Variant classification and interpretation

All variants were classified using guidelines from the American College of Medical Genetics and Genomics (ACMG) [12]. Our pathogenicity assessment integrated the following lines of evidence: (i) population frequency of the variant as estimated from the genome Aggregation Database (gnomAD)12 and 1000 Genomes13; (ii) scores from bioinformatic models including SIFT14 and PolyPhen-2 15; (iii) the level of evolutionary conservation at the protein and nucleotide sequence level, as estimated by the phyloP16 and GERP17 scores. When available, published literature and functional studies were also considered. Variants were classified into one of the following categories: pathogenic (P), likely pathogenic (LP), variant of uncertain significance (VUS), likely benign (LB) or benign (B). For the purpose of this study, only pathogenic variants were considered to be disease-causing.Segregation analysis was performed when family members were available. Novel variants (not reported in ClinVar, HGMD, or literature) were assessed using computational predictions and comparison with known pathogenic variants in the same gene. All identified pathogenic variants were absent or extremely rare (allele frequency < 0.01%) in gnomAD population databases, supporting their disease-causing role.

### 2.4 Statistical analysis

Descriptive statistics were provided for demographic and clinical characteristics. The means and standard deviations (SDs) or medians (range) of the continuous variables were provided after checking the normality of distribution by Shapiro-Wilk test. Frequencies (number) and percentages were provided for categorical variables. Mann-Whitney *U* test and Fisher's exact test were used for comparisons of continuous and categorical variables that were observed between VP and VN groups. To identify clinically relevant predictors of a positive genetic result, exploratory univariable analyses were performed for age at onset (continuous and categorized), family history of kidney disease, consanguinity, hematuria, extrarenal manifestations, and kidney transplantation. Given the limited sample size, these analyses were considered hypothesis-generating. A two-tailed *p* value less than 0.05 was regarded as statistically significant. All statistical analyses were conducted by using IBM SPSS Statistics version 26.0 (IBM Corp., Armonk, NY) and R version 4.2.0 (R Foundation for Statistical Computing, Vienna, Austria). Given the small sample size and lack of a control cohort, no formal rare variant burden analysis or multivariable modeling was performed; all genotype–phenotype comparisons were considered exploratory. Given the small sample size and single-center design, all statistical analyses and genotype–phenotype correlations were considered hypothesis-generating and should be interpreted with caution.

## 3 Results

### 3.1 Clinical characteristics

The study cohort consisted of 30 patients (18 males and 12 females) with biopsy proven FSGS. The mean age at genetic testing was 31.9 ± 11.9 years (range 14 to 56 years) with a median age of 28 years. The mean age at diagnosis was 27.0 ± 11.2 years (range 8 to 50 years) with a median age of 25 years. Family history of kidney disease was present in 25 (83.3%) patients. These features indicate that the cohort is highly enriched for familial and consanguineous cases with relatively early onset (mean age at diagnosis 27.0 ± 11.2 years), and therefore does not represent an unselected or general FSGS population. Consanguinity was observed in 20 (66.7%) patients. The clinical features present were proteinuria (93.3%), hypertension (76.7%), weight loss (66.7%) and hematuria (56.7%). Twelve (40%) patients had progressed to ESRD and required renal transplantation. Information on treatment and disease course was available for a subset of patients. Most patients received standard supportive therapy, including renin–angiotensin system blockade, and many were initially treated with corticosteroids according to local practice. Among those with available data, a substantial proportion had steroid-resistant disease, consistent with the high prevalence of presumed monogenic forms in this enriched cohort; however, detailed steroid response and precise time from onset to chronic kidney disease or kidney failure were not systematically recorded, limiting formal analysis of treatment response and progression. Table 1: Clinical and demographical data of the biopsied patient's Focal segmental glomerulosclerosis (FSGS) was identified in histopathology of all biopsied kidneys. FSGS NOS was the most common variant seen in 63.3% cases, while collapsing and hilar FSGS were seen in 3.3% cases each. Detailed demographic and clinical characteristics are presented in Table 1.

**Table 1:** Demographic and clinical characteristics of study population (*n* = 30).

Characteristic	Value
**Demographics**	
Age (years), mean ± SD	31.9 ± 11.9
Age (years), median (range)	28 (14–56)
Age of onset (years), mean ± SD	27.0 ± 11.2
Age of onset (years), median (range)	25 (8–50)
Male gender, n (%)	18 (60.0)

**Family and Genetic Background**	
Family history of kidney disease, n (%)	25 (83.3)
Consanguinity, n (%)	20 (66.7)

**Clinical Manifestations, n (%)**	
Proteinuria	28 (93.3)
Hypertension	23 (76.7)
Weight loss	20 (66.7)
Hematuria	17 (56.7)
Headache	9 (30.0)

**Outcomes**	
Kidney transplant, n (%)	12 (40.0)

### 3.2 Genetic findings

Next-generation sequencing revealed an adequate coverage to enable sequencing and variant detection in all 98 genes included in the gene panel. In a series of 30 patients who underwent gene panel sequencing, 10 mutations were identified in 10 patients (33.3% diagnostic yield). Mutations were found in 4 genes: COL4A4 in 3 (30%, *n* = 3) patients and JAG1 in 3 (30%, n = 3) patients, and in COL4A3 and NPHS2 each in 2 (20%, *n* = 2) patients. Frameshift (*n* = 8, 80%) mutations were the most common type of mutation, followed by missense (*n* = 1, 10%) and nonsense (n = 1, 10%) mutations. The zygosity distribution was 40% (4/10) homozygous and 60% (6/10) heterozygous. The mutations were inherited in an autosomal recessive (50%) or autosomal dominant (50%) pattern. Using the ACMG guidelines, all variants were classified as pathogenic. None of the variants were present in the gnomAD exomes or genomes or were present at very low frequency. Genetic testing results are summarized in Table 2.

**Table 2:** Genetic testing results in FSGS patients (*n* = 30).

Parameter	Value
Diagnostic yield, n (%)	10/30 (33.3)
Total pathogenic variants identified	10

Gene distribution, n (%)	
COL4A4	3 (30.0)
JAG1	3 (30.0)
COL4A3	2 (20.0)
NPHS2	2 (20.0)

Variant type, n (%)	
Frameshift	8 (80.0)
Missense	1 (10.0)
Nonsense	1 (10.0)

Zygosity, n (%)	
Homozygous	4 (40.0)
Heterozygous	6 (60.0)

Inheritance pattern, n (%)	
Autosomal recessive	4 (40.0)
Autosomal dominant	6 (60.0)

Novel variants identified, n (%)	2 (20.0)

Detailed variant information is presented in Table 3. Three patients had mutations in COL4A4: patient 1 had a homozygous frameshift mutation c.3109_3110delCT (p.Leu1037LysfsTer12), patient 14 had a heterozygous frameshift mutation c.1220delC (p.Pro407LeufsTer51) and patient 29 had a heterozygous missense mutation c.2590G > C (p.Gly864Arg). Two patients had mutations in COL4A3: patient 23 had a homozygous nonsense mutation c.2371C > T (p.Arg791Ter) and patient 13 had a heterozygous frameshift mutation c.1671_1672insG (p.Leu558AlafsTer26). Two un-related individuals (patients 11 and 15) carried the homozygous frameshift NPHS2 mutation c.102delA (p.Gly35AlafsTer64) confirming the presence of a founder mutation in this population. Three members of the same family (patients 4, 5 and 24) were heterozygous for the JAG1 frameshift mutation c.1713delC (p.Cys572ValfsTer3) showing clear familial segregation. A patient-level genotype–phenotype summary of the 10 variant-positive cases, including hematuria, extrarenal manifestations, and transplant status, is provided in Supplementary Table S2.

**Table 3:** Detailed description of pathogenic variants identified. Chromosome locations based on GRCh37/hg19. Hom = homozygous; Het = heterozygous; P = pathogenic; AR = autosomal recessive; AD = autosomal dominant; UN = uncertain/unknown; AF = allele frequency. “Absent” indicates variant not found in gnomAD v2.1.1/v3.1.2; “<0.01%” indicates extremely rare population frequency.

Patient	Gene	Chromosome	cDNA	Protein	Variant	Zygo-	ACMG	Inheri-	gnomAD
ID		Location	Change	Change	Effect	sity		tance	AF
1	COL4A4	chr2:227915732	c.3109_3110delCT	p.Leu1037LysfsTer12	Frameshift	Hom	P	AR	Absent
14	COL4A4	chr2:227958989	c.1220delC	p.Pro407LeufsTer51	Frameshift	Het	P	AD	<0.01%
29	COL4A4	chr2:227920787	c.2590G > C	p.Gly864Arg	Missense	Het	P	AD	Absent
23	COL4A3	chr2:228145303	c.2371C > T	p.Arg791Ter	Nonsense	Hom	P	AR	<0.01%
13	COL4A3	chr2:228135577	c.1671_1672insG	p.Leu558AlafsTer26	Frameshift	Het	P	AD	Absent
11	NPHS2	chr1:179544897	c.102delA	p.Gly35AlafsTer64	Frameshift	Hom	P	AR	Absent
15	NPHS2	chr1:179544897	c.102delA	p.Gly35AlafsTer64	Frameshift	Hom	P	AR	Absent
4	JAG1	chr20:10628614	c.1713delC	p.Cys572ValfsTer3	Frameshift	Het	P	AD	Absent
5	JAG1	chr20:10628614	c.1713delC	p.Cys572ValfsTer3	Frameshift	Het	P	AD	Absent
24	JAG1	chr20:10628614	c.1713delC	p.Cys572ValfsTer3	Frameshift	Het	P	AD	Absent

### 3.3 Genotype-phenotype correlations

**Fig. 1:**
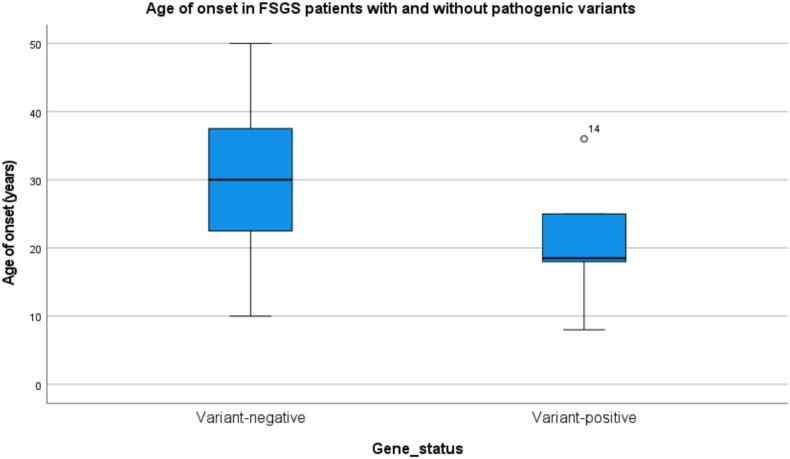
Age of onset in FSGS patients with and without pathogenic variants. Boxplots show age of disease onset in patients without pathogenic variants (variant-negative, *n* = 20) and with pathogenic variants in COL4A3, COL4A4, JAG1 or NPHS2 (variant-positive, *n* = 10). Variant-positive patients had significantly earlier onset compared to variant-negative patients (20.6 ± 7.2 vs 30.1 ± 11.6 years; Mann–Whitney *U* test, *p* = 0.022).

To explore the genotype-phenotype relationship, the clinical characteristics of patients with variant-positive and variant-negative mutations were compared (Table 4). Patients with pathogenic mutations had an earlier age of disease onset compared to Variant-negative patients (20.6 ± 7.2 years vs. 30.1 ± 11.6 years, respectively; *p* = 0.022; Fig. 1). However, the age at genetic testing (*p* = 0.128), gender (*p* = 0.706) and family history of kidney disease (*p* = 1.000) did not differ between the two groups. In the JAG1-positive family (patients 4, 5, and 24), extrarenal manifestations included ocular abnormalities and biochemical or imaging evidence of cholestatic liver disease, consistent with an Alagille-spectrum phenotype; however, the exact pattern and severity of extrarenal features differed between relatives, and not all components of the classic Alagille triad were systematically documented. These were most commonly observed in those with COL4A3/COL4A4 mutations (patients 2, 9) similar to the Alport syndrome spectrum disorders. Patient 6 had ocular and hepatic findings typical of Alagille syndrome due to JAG1 mutations (patients 4, 5, 24). Progression to ESRD and kidney transplantation was seen in 60% (6/10) of variant-positive patients versus 30% (6/20) of variant-negative patients (*p* = 0.230). Although the observation in this limited group of patients is intriguing, any conclusion should be interpreted with caution.

**Table 4:** Clinical characteristics comparison between variant-positive and variant-negative groups. Statistical tests: Mann-Whitney U test for continuous variables, Fisher's exact test for categorical variables.

Characteristic	Variant-Positive	Variant-Negative	*p*-value
	(n = 10)	(n = 20)	
Age at testing (years), mean ± SD	27.4 ± 10.2	34.2 ± 12.3	0.128
Age of onset (years), mean ± SD	20.6 ± 7.2	30.1 ± 11.6	0.022
Male gender, n (%)	7 (70.0)	11 (55.0)	0.706
Family history of kidney disease, n (%)	8 (80.0)	17 (85.0)	1.000
Consanguinity, n (%)	7 (70.0)	13 (65.0)	1.000
Proteinuria, n (%)	10 (100)	18 (90.0)	0.541
Hematuria, n (%)	7 (70.0)	10 (50.0)	0.448
Hypertension, n (%)	7 (70.0)	16 (80.0)	0.659
Kidney transplant, n (%)	6 (60.0)	6 (30.0)	0.230
Extrarenal manifestations, n (%)	4 (40.0)	2 (10.0)	0.162

### 3.4 Predictors of pathogenic variant detection

When patients were stratified by age at onset into three categories (<18 years, 18–30 years, and > 30 years), diagnostic yield was 40.0% (2/5), 46.7% (7/15), and 10.0% (1/10), respectively. This pattern supports that earlier disease onset enriches for monogenic FSGS and is consistent with prior observations in steroid-resistant nephrotic syndrome and genetic FSGS cohorts (Table 5). Earlier age at onset showed the strongest association with pathogenic variant detection, while extrarenal manifestations and hematuria were more frequent among variant-positive cases. Family history and consanguinity were common in both groups and therefore had limited discriminatory value within this enriched cohort (table6). A descriptive comparison of variant-positive cases by gene group is provided in Supplementary Table S3, showing distinct phenotype patterns across collagen IV-related disease, JAG1-associated syndromic disease, and NPHS2-associated podocytopathy. In an exploratory clinical triage approach, a simple rule based on age of onset <25 years, any extrarenal manifestation, or family history showed a sensitivity of 100.0% and a negative predictive value of 100.0% for detecting a pathogenic variant, whereas specificity was 20.0% and the positive predictive value was 38.5% in this cohort. These performance estimates should be interpreted cautiously, as they are derived from only 30 patients with high rates of family history and consanguinity and no external validation, and the rule should not yet be used as a standalone clinical decision tool until validated in larger, independent populations. (See Table 6.)

**Table 5:** Age-stratified diagnostic yield according to age of onset.

Age at onset group	Total patients	Variant-positive	Diagnostic yield (%)
<18 years	5	2	40.0
18–30 years	15	7	46.7
>30 years	10	1	10.0

**Table 6:** Clinical predictors of pathogenic variants.

Variable	Variant-positive (n = 10)	Variant-negative (n = 20)	Effect estimate	p-value
Age at onset (years), mean ± SD	20.6 ± 7.2	30.1 ± 11.6	NA	0.022
Onset <18 years	2 (20.0)	3 (15.0)	OR 1.42	1
Onset <25 years	7 (70.0)	6 (30.0)	OR 5.44	0.056
Male gender	6 (60.0)	12 (60.0)	OR 1.00	1
Family history of kidney disease	10 (100.0)	15 (75.0)	OR inf	0.14
Consanguinity	8 (80.0)	12 (60.0)	OR 2.67	0.419
Hematuria	9 (90.0)	8 (40.0)	OR 13.50	0.017
Extrarenal manifestations	5 (50.0)	5 (25.0)	OR 3.00	0.231
Kidney transplant	6 (60.0)	6 (30.0)	OR 3.50	0.139

Odds ratios were estimated in exploratory univariable analyses. Because of the limited sample size, confidence intervals should be interpreted cautiously and the analyses should be considered hypothesis-generating, in particular, the large odds ratio and nominal *p* value observed for hematuria arise from very small cell counts, without adjustment for other variables, and should be viewed as unstable, hypothesis-generating estimates rather than robust risk factors.

### 3.5 Novel variants

In this study, two novel frameshift variants were detected in COL4A4 (c.3109_3110delCT) and JAG1 (c.1713delC), which we classified as predicted pathogenic based on ACMG criteria and in silico evidence, although no functional validation is currently available this lead to expand the mutation spectrum of FSGS-associated genes. In Patient 1, the novel mutation COL4A4 c.3109_3110delCT (p.Leu1037LysfsTer12) was identified. This frameshift mutation resulted from a 2-bp deletion in the COL4A4 gene, leading to a premature stop codon at position 1048. Thus, the mutated gene was homozygous, as evidenced by the carrier status of the parents in the available members of the family, indicating an autosomal recessive inheritance pattern. The JAG1 variant c.1713delC (p.Cys572ValfsTer3) was found in three relatives (4, 5, 24). This frameshift deletion will result in premature protein truncation and an abnormal protein product. Mutations in the EGF-like domain 9 of JAG1, which contains binding sites for Notch and ligand, have typically been described as null mutations. The heterozygous genotype and apparent autosomal dominant inheritance with variability in expression in this family is consistent with Alagille syndrome. Neither variant was present in the gnomAD v2.1.1 or v3.1.2 datasets, ClinVar or published literature. SIFT, PolyPhen-2 and CADD predictions suggest that both variants are deleterious. Submissions have been made to ClinVar. Among the ten variants, eight corresponded to previously reported or ClinVar-annotated pathogenic alleles, whereas two (COL4A4 c.3109_3110delCT and JAG1 c.1713delC) were newly identified in this cohort and are described here as predicted pathogenic.

## 4 Discussion

Our cohort of consecutive patients had a high prevalence of family history and consanguinity and had a diagnostic yield of 33.3%. This pattern indicates that the series is heavily enriched for familial and early-onset FSGS, likely inflating the proportion of monogenic disease relative to unselected or sporadic FSGS populations, and directly explaining the relatively high diagnostic yield observed. Several of the FSGS cases harbored mutations in genes that have previously been reported in other hereditary forms of kidney disease such as COL4A3, COL4A4 and JAG1 leading to phenotypic overlap with other conditions within the Alport syndrome spectrum and Alagille syndrome [13], [14], [15], [16]. In conclusion, gene panel sequencing is a useful tool in the diagnosis of FSGS and can provide important information regarding prognosis and genetic counseling for families affected with this condition, because our cohort comprised only 30 patients and lacked an independent control group, our findings should be considered preliminary. Larger, multicenter studies will be required to confirm these observations and refine clinical indications for testing.

### 4.1 Diagnostic yield and gene Spectrum

Our diagnostic yield of 33.3% falls within the range of 10–40% previously reported in genetic studies of patients with FSGS [5], [17]. Several factors may have contributed to achieving a higher diagnostic yield in this study, including a high rate of family history (83.3%) and consanguinity (66.7%). Taken together, these features define our series as a familial, early-onset, highly consanguineous FSGS cohort enriched for monogenic disease, rather than a representative sample of sporadic or secondary FSGS. In addition, the use of a broad 98 gene panel for sequencing which is likely to encompass the majority of known genes that have been implicated in FSGS pathogenesis and the fact that this study included patients with both pediatric and adult forms of FSGS.

While mutations in genes encoding proteins of the podocyte such as NPHS1, NPHS2, and WT1 as well as other genes have been commonly identified in children with SRNS, in our series of adult SRNS, mutations in type IV collagen genes COL4A3 and COL4A4 were found in 50% of mutations identified in our series of adults, followed by 30% for JAG1 mutations and 20% for NPHS2 mutations [18], [19], [20]. Indeed, mutations in COL4A3/COL4A4 are currently the most commonly identified mutations in adults with adult-onset FSGS [14], [21]. The differential diagnosis between FSGS and Alport syndrome can sometimes be challenging and, with an increasing number of publications reporting adult patients initially diagnosed with FSGS but later reclassified into the spectrum of Alport syndrome following the identification of mutations in COL4A3/COL4A4 and/or COL4A5 genes [13], [22], [23]. Our findings are consistent with recent adult FSGS cohorts in which COL4A3/COL4A4 and podocyte genes account for a substantial fraction of monogenic cases and support emerging recommendations for broader genetic testing in FSGS.

### 4.2 COL4A3 and COL4A4 variants

Type IV collagen genes encode the proteins that constitute the glomerular basement membrane. Mutations in COL4A3, COL4A4 and COL4A5 have traditionally been associated with Alport syndrome. Recently, mutations in these genes have been also described as a possible cause of a fraction of focal segmental glomerulosclerosis (FSGS) cases [14], [22], [24]. In our series of cases, mutations in COL4A3 and COL4A4 genes were found in 5 patients (50% of solved cases). Two patients had homozygous truncating mutations characteristic of the autosomal recessive form of Alport syndrome, while three patients harbored heterozygous mutations indicative of either an autosomal dominant disease or digenic inheritance. All the patients in this study had proteinuria and progressive renal failure. Other systemic symptoms were found in 40% of the cases including hearing loss and ocular abnormality. The clinical spectrum in this study fits well with the concept of a continuous spectrum from isolated hematuria (thin basement membrane nephropathy) to FSGS to classic Alport syndrome according to the different mutations and genotypes. The predominance of COL4A3/COL4A4 variants among solved cases in the present study is concordant with growing evidence that collagen IV gene defects are among the most frequent genetic explanations for adult-onset and familial FSGS. Several groups have shown that COL4A3-COL4A5 variants may present with proteinuria and segmental sclerosis, sometimes without classic early recognition of Alport syndrome, leading to diagnostic overlap between inherited basement membrane disease and primary podocytopathy. The combination in the current cohort of proteinuria, hematuria, renal impairment, and extrarenal manifestations in a subset of collagen IV-positive patients supports this spectrum concept and argues for deliberate review of Alport-spectrum clues whenever COL4A3/COL4A4 variants are identified [25], [26].

### 4.3 JAG1 variants and Alagille syndrome

Three related individuals (4, 5, 24) harbored the same heterozygous JAG1 frameshift mutation, c.1713delC (p.Cys572ValfsTer3), which was also identified in the kidneys (FSGS) and ocular and liver tissues. JAG1 encodes a Notch ligand critical for the development of several organ systems. Function–losing mutations in JAG1 lead to Alagille syndrome, characterized by bile duct hypoplasia, congenital heart defects, skeletal abnormalities, ocular dystopia, and distinctive facial features [27], [28].

Intrarenal abnormalities in Alagille syndrome have been reported more frequently in the literature and can include congenital defects like renal dysplasia and vesicoureteral reflux as well as glomerulopathies [15], [29]. Identification of JAG1 mutations in an individual with FSGS and apparent multisystem disease due to Alagille syndrome further broadens the clinical presentation of the syndrome and suggests that patients with FSGS and multisystem disease should be fully evaluated and genetically tested. The JAG1 findings are particularly notable because they support the view that adult renal presentations of Alagille syndrome may be underrecognized. Recent reviews indicate that kidney involvement can be present in a substantial proportion of patients with Alagille syndrome and may occasionally be a presenting feature, while JAG1-Notch signaling is also relevant to kidney development and vascular remodeling. In our family, the clustering of FSGS with ocular and hepatic abnormalities in JAG1 carriers, together with the absence of similar extrarenal findings in non-carriers, supports the interpretation of this variant as an Alagille-spectrum lesion, although incomplete documentation of cardiac, skeletal, and facial features means that a full syndromic characterization was not possible. The clustering of FSGS with ocular and hepatic abnormalities in this family therefore strengthens the interpretation that at least some biopsy-proven FSGS cases represent syndromic kidney disease rather than isolated glomerular pathology.

### 4.4 NPHS2 variants and recessive FSGS

Two unrelated probands (11 and 15) were homozygous for a frameshift mutation, c.102delA (p.Gly35AlafsTer64) in NPHS2. As we have seen previously, NPHS2 encodes the protein podocin which is crucial component for the slit diaphragm of the glomerulus. Mutations in this gene have been described in cases of congenital SRNS (early onset) and familial FSGS [8], [30], [31].Interestingly, in this case the mutation was described in two different consanguineous families pointing to a possible founder effect and underlying a different mutation spectrum in different populations. Children with mutations in NPHS2 gene develop early-onset nephrotic syndrome with resistance to corticosteroids, and progress to ESRD and require a renal transplant [32]. Our two patients also had similar clinical features and perform renal transplant. Another aspect that needs to be considered is that recurrent FSGS after renal transplant is uncommon in patients with NPHS2 mutations, so genotype diagnosis is helpful for renal transplant counseling. The repeated identification of the same homozygous NPHS2 frameshift variant in two unrelated consanguineous families raises the possibility of a regional founder allele. This observation has practical implications because NPHS2-related disease is classically associated with steroid-resistant nephrotic syndrome and, importantly, with a very low risk of post-transplant recurrence compared with idiopathic forms. In a recent cohort of patients with biallelic NPHS2 pathogenic variants, recurrence after kidney transplantation was extremely uncommon, supporting the value of genotype-based counseling for transplant planning and donor assessment.

When the variant-positive cases were grouped by gene, distinct genotype–phenotype patterns became apparent. Patients with COL4A3/COL4A4 variants tended to have earlier onset, hematuria, and a higher burden of hearing abnormalities, consistent with collagen IV-related basement membrane disease. In contrast, NPHS2-positive patients showed a recessive podocytopathy with early-onset disease and progression to ESRD but without syndromic extrarenal involvement, while the JAG1-positive family combined FSGS with prominent ocular findings in keeping with Alagille-spectrum disease. These observations illustrate that an identical biopsy label of FSGS can result from basement membrane disorders, slit-diaphragm defects, or multisystem developmental syndromes.

### 4.5 Genotype–phenotype correlations

The most consistent genotype–phenotype pattern observed in this cohort was an earlier age at disease onset among variant-positive patients compared with variant-negative patients (20.6 ± 7.2 vs. 30.1 ± 11.6 years; *p* = 0.022). In exploratory univariable analysis, onset before 25 years showed the strongest categorical association with a positive genetic result, while hematuria and extrarenal manifestations were also more frequent among variant-positive cases; however, these findings should be interpreted as descriptive and hypothesis-generating given the small sample size and absence of multivariable modeling.

Patients with COL4A3 or COL4A4 variants predominantly showed renal features compatible with collagen IV–related nephropathy, including proteinuria and hematuria, with variable age at onset. In this cohort, these cases were descriptively more consistent with a collagen IV–related disease spectrum than with primary FSGS alone, although the sample size was too small to define a distinct phenotype or make robust clinicopathological reclassification. ESRD and transplantation were observed in some patients with collagen IV gene variants, but these outcome data should be interpreted cautiously because of the limited number of cases.

Patients with NPHS2 variants presented with earlier-onset disease, in keeping with recessive podocytopathy and monogenic steroid-resistant nephrotic syndrome patterns reported in the literature. The phenotype in these patients was primarily renal, without a broader syndromic presentation in the available dataset. Although adverse renal outcomes were observed in some cases, treatment response and long-term renal progression were not systematically assessed, limiting interpretation of prognostic differences.

Patients with JAG1 variants showed renal disease accompanied by extrarenal manifestations, supporting a phenotype compatible with an Alagille-spectrum disorder rather than isolated primary FSGS. However, this interpretation remains provisional because formal syndromic evaluation, imaging, and liver-focused assessment were not systematically performed in all cases. Accordingly, these findings should be regarded as descriptive and supportive of further evaluation rather than definitive reclassification [18], [33], [34].

#### 4.5.1 .Clinical predictors and triage implications

Family history of kidney disease and consanguinity were common in both variant-positive and variant-negative groups, which reduced their discriminatory value within this highly enriched cohort. By contrast, earlier onset and syndromic or extrarenal features appeared to be more informative for identifying patients more likely to have a detectable pathogenic variant. A simple clinical triage approach based on age at onset, extrarenal manifestations, and family history may help prioritize patients for gene-panel testing in resource-limited settings; however, this should be viewed as a preliminary, data-driven observation rather than a standalone clinical decision tool, and it requires validation in larger, independent cohorts. Apparent associations such as the high odds ratio for hematuria must therefore be interpreted with caution, as they are based on small numbers, wide but unreported confidence intervals, and univariable analyses only.

#### 4.5.2 ESRD and transplantation

We observed numerical differences in ESRD and transplantation across some gene groups, but the small number of variant-positive patients precludes robust statistical inference. Therefore, any apparent differences in renal outcomes between variant-positive and variant-negative patients, or across gene-specific subgroups, should be interpreted cautiously and considered exploratory rather than confirmatory.

[18], [33], [34]. Due to limited sample size and lack of multivariable modeling, all genotype–phenotype associations should be viewed as exploratory and hypothesis-generating rather than confirmatory. In settings where access to sequencing is limited, a simple rule based on age at onset and extrarenal findings may help prioritize testing, although formal validation is needed before clinical implementation.

### 4.6 Novel variants and population-specific spectrum

Here we describe two previously unreported frameshift variants, COL4A4 c.3109_3110delCT and JAG1 c.1713delC, which we classified as predicted pathogenic based on ACMG criteria and in silico evidence, although no functional validation is currently available. Neither variant was present in the gnomAD database, and segregation analysis demonstrated that they were present in affected family members but absent in unaffected members and thus were predicted to be deleterious by bioinformatics tools. The identification of population-specific variants that may not be present in databases highlights the need for genetic studies in multiple populations to fully elucidate the mutational spectrum of genes and facilitate variant interpretation. Two novel frameshift variants were detected in COL4A4 (c.3109_3110delCT) and JAG1 (c.1713delC), which we classified as predicted pathogenic based on ACMG criteria and in silico evidence, although no functional validation is currently available.

High consanguinity rate in our cohort (66.7%) gave us an opportunity to investigate the role of rare recessive mutations in the homozygous state as well as the effect of novel mutations that would not have been identified or recognized in out-bred populations due to carriers being heterozygous [11].

### 4.7 Clinical implications

Our study has a number of clinical implications. First, we believe that genetic testing may be considered as part of the evaluation of FSGS, particularly in selected young patients with FSGS, those with consanguinity or a family history, and those with extrarenal manifestations. Second, given the genetic heterogeneity of FSGS, we believe that the use of gene panels covering all genes of interest, including COL4A3, COL4A4, COL4A5, JAG1, and major podocyte genes, is more appropriate than sequencing individual genes. When a pathogenic mutation is confirmed in a patient with FSGS, it has a number of clinical implications. Patients with a confirmed monogenic form of FSGS are unlikely to benefit from immunosuppressive therapy and therefore can be safely spared the potential toxic effects of immunosuppressive therapy. The genetic diagnosis can also be used for genetic counseling and for cascade testing and family reproductive planning. Understanding the underlying genetic mutation in a patient with FSGS can also affect posttransplant management of these patients, because the risk of recurrence of FSGS after transplantation is very different in immune mediated forms versus monogenic forms of FSGS. These findings support the potential value of gene-panel testing in carefully selected young or syndromic FSGS patients, particularly in consanguineous populations, but our data are insufficient to recommend gene-panel testing as a universal first-line approach.

### 4.8 Unsolved cases and future discovery

Despite using a 98-gene renal panel, two-thirds of our patients (20/30, 66.7%) remained genetically unresolved. Several factors may explain this diagnostic gap, including structural variants (e.g., copy-number variants), deep intronic or regulatory variants not captured by the panel design, and pathogenic variants in genes not yet associated with FSGS or not included in our panel. Variant-negative patients may harbor variants in genes not included on the panel, or oligogenic contributions that are difficult to resolve using targeted sequencing alone. This is especially relevant in consanguineous populations, where extended runs of homozygosity may enrich for recessive alleles in genes that are not yet recognized as causes of FSGS. Exome or genome sequencing in unresolved cases may therefore provide additional diagnostic yield and could identify population-specific disease genes. Whole-exome or whole-genome sequencing, ideally combined with robust CNV detection, would likely increase the diagnostic yield in unresolved cases by capturing variants outside the targeted regions and enabling discovery of novel disease genes. Nevertheless, such approaches remain costly and may not yet be widely available in our setting.

### 4.9 Limitations

This study has several limitations. First, the cohort size was modest, which limited statistical power and means that the exploratory predictor analyses should be interpreted cautiously. Second, not all family members were available for segregation analysis, restricting formal assessment of inheritance and penetrance in some families. Third, the targeted panel design, while clinically efficient, may miss copy-number variants, deep intronic variants, structural rearrangements, and pathogenic variants in genes not yet associated with FSGS. Fourth, functional validation was not available for the two novel variants, so pathogenicity inference relied on ACMG criteria, population rarity, predicted loss-of-function effect, and available segregation data. Finally, our cohort was highly enriched for patients with a positive family history (83.3%) and consanguinity (66.7%), reflecting referral patterns and local epidemiology. This enrichment likely increased the genetic diagnostic yield and may have artificially increased the apparent sensitivity of the clinical triage rule. Therefore, our findings should not be extrapolated to unselected FSGS populations, adults with secondary FSGS, or settings with lower consanguinity rates without further validation. We did not include ancestry-matched controls or perform rare variant burden testing, so our findings cannot address enrichment at the gene or pathway level and are limited to within-cohort diagnostic yield and phenotype correlations. Future multicenter studies combining deeper phenotyping, pathology re-review, extended family studies, and exome or genome sequencing will be important to validate these findings and refine testing strategies. Also, we did not re-review all biopsies with electron microscopy or specialized staining, and systematic extrarenal evaluation was not performed in all patients; therefore, our proposed reclassification into Alport spectrum or Alagille spectrum remains provisional and should be confirmed by comprehensive clinicopathological correlation in future studies.

## 5 Conclusion

The study demonstrates a diagnostic yield of 33.3% FSGS patients using a targeted gene panel and COL4A3/COL4A4 and JAG1 genes were the most mutation bearing and are associated with Alport and Alagille spectrum of disorders. Patients with mutations were diagnosed at an earlier age and we believe genetic testing to be of benefit in patients with early presentation, consanguinity and a family history.

## CRediT authorship contribution statement

**Lava I. Ahmed:** Writing – original draft, Validation, Project administration, Investigation, Data curation, Conceptualization. **Dlnya A. Mohammed:** Supervision. **Dana Ahmed Sharif:** Supervision.

## Ethics approval

This study was approved by the institutional review board and conducted in accordance with the Declaration of Helsinki. Written informed consent was obtained from all participants or their legal guardians.

## Funding

This research received no specific grant from funding agencies in the public, commercial, or not-for-profit sectors.

## Declaration of competing interest

The authors declare no conflicts of interest.

## Data Availability

The datasets generated and analyzed during this study are available from the corresponding author upon reasonable request, subject to appropriate ethical approvals and data sharing agreements. The ClinVar accession numbers for this submission are SCV007538964 – SCV007538970.
