# Supplementary material for: Pathogenic variants in COL4A3, COL4A4, JAG1, and NPHS2 genes in focal segmental glomerulosclerosis: Insights from targeted gene panel sequencing
Source: Mol Genet Metab Rep. 2026 Jun 15;48:101330. doi: 10.1016/j.ymgmr.2026.101330 (PMC13285704; doi:10.1016/j.ymgmr.2026.101330)
Supplement: Supplementary material [file mmc1.docx]

**Supplementary Table S1.** Complete list of 98 genes included in the targeted next-generation sequencing panel. The panel was designed to cover genes associated with focal segmental glomerulosclerosis (FSGS), steroid-resistant nephrotic syndrome, glomerular basement membrane disorders, and ciliopathies. Genes are grouped by functional category (glomerular basement membrane, podocyte/cytoskeleton, developmental transcription factors, and ciliopathy and related genes). The panel was designed using Ion AmpliSeq Designer (version 6.2) for sequencing on the Ion S5 platform (Thermo Fisher Scientific).

| **Category** | **Gene symbol** |
| --- | --- |
| Glomerular basement membrane | COL4A3; COL4A4; COL4A5 |
| Podocyte / slit diaphragm / cytoskeleton | NPHS1; NPHS2; ACTN4; INF2; MYO1E; TRPC6; APOL1 |
| Renin–angiotensin / kidney function | REN; UMOD |
| Developmental / transcription factors / GBM-related | HNF1A; HNF1B; HNF4A; PAX2; LMX1B; JAG1; SALL1; SIX1; SIX5; WT1 (if present in your full list) |
| Ciliopathy and nephronophthisis genes / IFT / transition zone | KIF14; NOTCH2; NPHP1; NPHP3; NPHP4; SDCCAG8; IFT172; TMEM237; TTC21B; WDPCP; ARL13B; ARL6; DZIP1L; IQCB1; LZTFL1; WDR19; C5orf42; CEP120; AHI1; DCDC2; WDR19; GLIS2; CEP290; INPP5E; INVS; TMEM67; MKS1; TMEM138; TMEM216; TCTN1; TCTN2; TCTN3; KIAA0586; B9D1; TMEM231 |
| Bardet–Biedl and related | BBS1; BBS2; BBS4; BBS5; BBS7; BBS9; BBS10; BBS12; BBIP1; TTC8; MKKS |
| Coenzyme Q and glycosylation / ER‑related | COQ2; COQ6; PDSS1; PDSS2; GANAB; PRKCSH; ALG8; ALG9 |
| PKD / CAKUT / structural kidney disease | PKD1; PKD2; PMM2; OFD1 |
| Other glomerular / signaling genes | GLIS3; CRB2; VHL; LRP5; ZNF423; NEK8; XPNPEP3; RPGRIP1 |

Supplementary table 2: Data are shown for the 10 patients with confirmed pathogenic variants in COL4A3, COL4A4, JAG1, or NPHS2. Hematuria and extrarenal manifestations are based on clinical records at presentation; ‘Clinical interpretation’ reflects the likely disease category (collagen IV-related disease, recessive podocytopathy, or Alagille-spectrum syndromic FSGS).

| Patient ID | Gene | Variant | Zygosity | Age at onset (years) | Hematuria | Hearing loss | Ocular findings | Hepatic/cardiac findings | Transplant/ESRD | Clinical interpretation |
| --- | --- | --- | --- | --- | --- | --- | --- | --- | --- | --- |
| 13 | COL4A3 | c.1671_1672insG | heterozygous | 18 | Yes | No | No | No | Yes | Collagen IV-related FSGS |
| 23 | COL4A3 | c.2371C>T | homozygous | 8 | No | Yes | No | No | No | Collagen IV-related FSGS |
| 1 | COL4A4 | c.3109_3110delCT | homozygous | 18 | No | Yes | No | No | Yes | Collagen IV-related FSGS |
| 14 | COL4A4 | c.1220delC | heterozygous | 36 | Yes | No | No | No | No | Collagen IV-related FSGS |
| 29 | COL4A4 | c.2590G>C | heterozygous | 25 | Yes | No | No | No | No | Collagen IV-related FSGS |
| 4 | JAG1 | c.1713delC | heterozygous | 19 | No | No | Yes | No | Yes | Alagille-spectrum FSGS |
| 5 | JAG1 | c.1713delC | heterozygous | 25 | No | Yes | Yes | No | Yes | Alagille-spectrum FSGS |
| 24 | JAG1 | c.1713delC | heterozygous | 22 | Yes | No | Yes | No | No | Alagille-spectrum FSGS |
| 11 | NPHS2 | c.102delA | homozygous | 17 | No | No | No | No | Yes | Recessive podocin-related FSGS |
| 15 | NPHS2 | c.102delA | homozygous | 18 | Yes | No | No | No | Yes | Recessive podocin-related FSGS |

| Gene group | n | Age of onset, mean ± SD (years) | Hematuria, n (%) | Hearing loss, n (%) | Ocular findings, n (%) | Hepatic findings, n (%) | Transplant/ESRD, n (%) | Family history, n (%) | Consanguinity, n (%) |  |
| --- | --- | --- | --- | --- | --- | --- | --- | --- | --- | --- |
| COL4A3/COL4A4 | 5 | 21.0 ± 10.3 | 3 (60.0) | 2 (40.0) | 0 (0.0) | 0 (0.0) | 2 (40.0) | 5 (100.0) | 3 (60.0) |  |
| JAG1 | 3 | 22.0 ± 3.0 | 1 (33.3) | 1 (33.3) | 3 (100.0) | 0 (0.0) | 2 (66.7) | 3 (100.0) | 1 (33.3) |  |
| NPHS2 | 2 | 17.5 ± 0.7 | 1 (50.0) | 0 (0.0) | 0 (0.0) | 0 (0.0) | 2 (100.0) | 2 (100.0) | 1 (50.0) |  |

Supplementary table 3: Data are summarized for the 10 patients with confirmed pathogenic variants grouped by gene. Percentages are calculated within each gene group
